# Letter to the editor regarding “safety of safety evaluation of pesticides: developmental neurotoxicity of chlorpyrifos and chlorpyrifos-methyl” by Mie et al. (environmental health. 2018. 17:77)

**DOI:** 10.1186/s12940-019-0454-x

**Published:** 2019-03-15

**Authors:** Daland R. Juberg, Alan M. Hoberman, Sue Marty, Catherine A. Picut, Donald G. Stump

**Affiliations:** 1 Human Health Science Policy, Corteva Agrisciences, Indianapolis, IN USA; 2 Global Developmental, Reproductive and Juvenile Toxicology, Charles River Laboratories, Horsham, PA USA; 3 Toxicology & Environmental Research and Consulting, The Dow Chemical Company, Midland, MI USA; 4 Charles River Laboratories, Durham, NC USA; 5 Charles River Laboratories, Ashland, OH USA

January 24, 2019

Dear Drs. Grandjean and Ozonoff, Editors-in-Chief, Environmental Health.

We are providing the following perspectives in response to a recently published Commentary in *Environmental Health* titled “Safety of Safety Evaluation of Pesticides: developmental neurotoxicity of chlorpyrifos and chlorpyrifos-methyl” by Mie et al. [15]. We, the undersigned, disagree with specific technical points, assertions, and assumptions and are providing the following scientific perspectives in defense of the design, conduct, and interpretation of the developmental neurotoxicity studies conducted for both chlorpyrifos and chlorpyrifos-methyl.

## Summary

A commentary in *Environmental Health* titled “Safety of Safety Evaluation of Pesticides: developmental neurotoxicity of chlorpyrifos and chlorpyrifos-methyl” by Mie et al. [15] erroneously suggests that developmental neurotoxicity studies conducted for chlorpyrifos and chlorpyrifos-methyl and then submitted to regulatory authorities were misleading and impaired the authorities’ ability to perform a valid evaluation. We will address the specific technical points, assertions and assumptions individually and demonstrate why the chlorpyrifos study - conducted more than 20 years ago - was reviewed and accepted by global regulatory authorities, including the U.S. Environmental Protection Agency, Australian Pesticides and Veterinary Medicines Authority, Health Canada Pest Management Regulatory Agency and the European Union. Labeled uses of chlorpyrifos rest on five decades of experience in use, health surveillance of manufacturing workers and applicators, and more than 4000 studies and reports examining the product in terms of health, safety and the environment. We will also respond to the allegations made about the chlorpyrifos-methyl study which was conducted more recently.

### Brain weight

Mie et al. [15] challenge the chlorpyrifos DNT study by stating that *“For high dose pups, the test laboratory reported a significant reduction of total brain weight and the dimensions of several brain regions on PND 11, but not on PND 65. The test lab argued that these observed effects do not indicate DNT…To support this interpretation, the test laboratory calculated that the average effect on all brain regions is similar to the effect on brain weight…Accordingly, the EPA has identified this analytical approach as an inappropriate and inconclusive manipulation of the data, but a correction was apparently not requested from the pesticide producer submitting the report.”*

The methodology used in the DNT studies was sound. The highest dose tested in the chlorpyrifos DNT study produced an appropriate level of maternal toxicity, verifying the use of adequate dose levels in this study. The toxicity observed (reduction in bodyweight gain and food consumption, adverse clinical observations, reduction in cholinesterase levels) did not interfere with the production of offspring but did result in a reduction in pup body weight and as noted by Mie et al. [15], a reduction in brain weight and the size of several brain regions in PND 11 pups but not in PND 65 adult rats.

In addition to the reduction in weight and measurements of brain areas, the study design included a functional evaluation (i.e., behavioral assessments) in the offspring post weaning and a neurohistopathological assessment of the brain at two ages (i.e., PND 11 and 65) by a board certified neuropathologist.

In the chlorpyrifos study, effects in the high dose pups were only observed on the weights and brain area measurements and these effects were not apparent when the pups grew up (at PND 65). The neurohistopathology of the CNS and peripheral nervous system and behavioral assessments were unaffected at the highest dose tested. When results such as observed in this study are produced, there could be several explanations. What the “testing laboratory was arguing” was supported by previously published articles [5, 6, 10] that demonstrated effects on brain weight and the size of various areas of the brain, without any other evidence of damage to the nervous system, is often due to undernutrition. This undernutrition would be expected in offspring of mothers who were growth retarded and food deprived during gestation. In addition to the literature references, a calculation of the % reduction in brain weight to the % reduction in the size of various brain regions was conducted to further support this theory of undernutrition causing the effects in the highest dose group.

The real difference in opinion from the evaluation of the study results was in the interpretation of statistically significant reductions in the size of selected regions of the brain in the lower dose groups on PND 11. These differences were not apparent in the middle dose group of the adult rats (PND 65), the tissues were histologically normal at all doses, and there were no functional deficits at any dose, so it was argued that no developmental neurotoxicity was occurring at any dose level but clearly none was occurring at the middle dose level.

### U-shaped dose-response

Mie et al. [15] assert that *“the proper way to demonstrate the absence of a sensitive target region is to express each brain regional measure relative to brain weight...”* Further they contend that *“the U-shaped dose-response relationship in Fig 1b is consistent with an effect of chlorpyrifos on the cerebellum height at low, mid and high dose, and on overall brain weight at high dose only…hence indicating the presence of DNT at all dose levels tested.”*

Adjusting the regional brain measurement to the brain weight to obtain a relative measurement of the brain region (the actual measurement adjusted for the brain weight) is an appropriate method for comparison to be made across groups when no other methodological variables have affected either the regional brain measurements or the brain weights. In the chlorpyrifos study, all brain weights were obtained in a comparable pattern in terms of the time post fixation. For the PND 11 brains, there was a difference in the timing to embedding of the tissues postfixation for the control and high dose group pups, compared to the low and mid dose groups. A difference of 39 days occurred in between these processes. This difference in processing time could explain the apparent effect in the low and mid dose groups and the apparent U-shaped response curve. Therefore, the new calculations for the cerebellum height measurement data and the relationship to the brain weights as presented in Mie et al. [15] are flawed.

This developmental neurotoxicity study was one of the first conducted under the DNT guideline and morphometric measurements were a relatively new assessment. At the time this study was conducted (i.e., 1998), it was standard practice for histopathology to first evaluate the control and high dose groups and if a difference was observed then the next lower dose group would be evaluated. The additional processing of the neural tissue for the lower dose groups occurred over a month after the original processing. These tissues had additional time in fixative and we have found that additional shrinkage of the tissue does occur. Based on the difference in fixation time, differences in the cerebellar height would be expected and the apparent U-shaped dose response is more than likely an expected artifact.

### Pup exposure regimen

Mie et al. [15] note that “*The brain growth spurt occurs mainly postnatally in rats but prenatally in humans. Accordingly, a dosing scheme maintaining an exposure of neonatal pups equivalent to continuous in utero exposure in humans would be most relevant for addressing effects during this vulnerable developmental stage.”*

There are two issues with this statement – confusion with respect to the brain growth spurt and confounding effects that are introduced by dosing during the early neonatal period. First, as noted by Dobbing and Sands [7], who wrote extensively on the brain growth spurt across species, the rat brain growth spurt begins prenatally, but is primarily postnatal, continuing to approximately postnatal day 17–21. The human brain growth spurt also spans both the prenatal and postnatal period with a greater proportion of the growth spurt in the postnatal period (not prenatal as suggested by the authors) and continuing to approximately 18 months of age.

Secondly, while it may be desirable to continue consistent dosing after birth, it is difficult to maintain in utero exposure levels during the postnatal period in rat pups. To do this, direct dosing of neonatal pups would be required, which is not a standard requirement of DNT studies and requires careful consideration per the OECD Developmental Neurotoxicity Test Guideline [17]. Direct dosing of neonatal rat pups is technically difficult and stressful for the rat pups. Furthermore, pup internal doses should be titrated to fetal dose levels which would require consideration of the pharmacokinetic profile in fetal versus postnatal and adult rats, including differences due to route of administration (e.g., oral to pups vs. via blood secondary to maternal metabolism and placental transfer in fetuses), timing of exposure (e.g., bolus dose in the pup vs. maternal absorption and distribution to fetuses), etc. For these reasons, pups are generally not given direct doses of test material when lactational transfer occurs. In the case of the chlorpyrifos DNT study, lactational exposure ensured continuous exposure during the early postnatal period, which is an equivalent neurodevelopmental stage to human in utero.

### Blood Chlorpyrifos levels

Mie et al. [15] state that: *“A parallel study sponsored by the same company shows that the chlorpyrifos concentration in the blood from nursing pups decreased substantially, compared to fetal blood levels, because only a small fraction of the continued maternal exposure is transferred via milk.”*

Chlorpyrifos, which is lipophilic (K_ow_ ~ 4.7), partitions readily into milk and in rodent studies, achieves considerably greater levels in milk than seen in maternal blood [13]. Chlorpyrifos levels in milk change over time with changes in milk lipid levels, achieving high levels on lactation day/postnatal day 1 (3022 ng chlorpyrifos/g milk with a 5 mg/kg/day dose administered to the dam) and lower levels (1534 ng/g milk) on lactation day/postnatal day 5 [13]. This is consistent with the post-gestational decrease in maternal blood levels of chlorpyrifos. Higher levels of maternal chlorpyrifos at the end of gestation are related to the increase in plasma lipid levels that occurs during the latter part of gestation. The increased partitioning of chlorpyrifos into plasma lipids in the pregnant dam results in less chlorpyrifos in other tissue compartments [12]. Furthermore, gavage dosing with corn oil also increases plasma lipid levels, which further increases chlorpyrifos partitioning into plasma. Lastly, it is worth noting that blood levels in the Mattsson et al. [13] paper reflect single time points and do not include area-under-the-curve (AUC) values to indicate total exposure over time.

### Statistical approaches

Mie et al. [15] state that “*We also note post-hoc changes to the statistical protocol as well as an unusually low cut-off for statistical significance in the study report, although a justification was not provided.”*

The post-hoc changes to the statistical protocol were suggested by the EPA in their 1998 review. The original statistical scheme was well-defined and appropriate but not commonly conducted. This statistical analysis scheme and the statistical significance cut-off level of (α = 0.02) was based on the recommendations proposed by Tukey et al. [20]. The supplemental additional statistical analyses conducted on the morphometric measurements were done in response to the 1998 EPA data evaluation record (DER) and in consultation with the EPA.

### Purpose of positive control agent

Mie et al. [15] also challenge the test facility and its ability *“to detect neurobehavioral effects of elevated developmental exposure to lead nitrate, although lead is a confirmed developmental neurotoxicant at very low doses.”*

The reason for the lack of neurotoxic effects when lead was administered was likely due to the use of lead nitrate rather than lead acetate and the dose of lead nitrate used. The administration of lead nitrate produced maternal (e.g., clinical observations, reduced feed consumption) and developmental toxicity (e.g., reduced litter size, pup weights and food consumption). The absence of any developmental neurotoxicity may have been due to a lack of exposure or the fact that the affected pups may have died in utero. The purpose of the positive control study is to ensure that the technical staff and equipment being used can demonstrate a difference from negative control. Other positive control data generated at the testing facility using trimethyl tin, amphetamine and acrylamide were included in the study report, and demonstrated adequate training of the technical staff and appropriate test procedures.

### Missing data points

Mie et al. [15] note that in the developmental neurotoxicity (DNT) study of chlorpyrifos-methyl in rats performed by WIL Research in [22] (WIL-406032) “*40 out of 80 data points are missing in the report, with an explanation provided only for 40% of the missing data*”. This DNT study was also criticized for the pathologist concluding that “*the absence of these few data points affecting cerebellum of females at PND 72 does not affect the interpretation of the study”.*

It is understandable how missing data points can raise concern in a GLP study, but with DNT studies, absent morphometric data points are to be expected. First, the use of the term “missing” data by Mie et al. [15] is misleading. In DNT studies, there is a deliberate action to exclude certain measurements from any one section of brain, when the section is non-homologous or the measurement would be affected by *processing* artifact. In fact, if a pathologist fails to exclude certain measurements from less than ideal sections, the data could be deemed unreliable. Garman [9] acknowledges that some measurements must be discarded when he stated that “some institutions … assign greater than 10 rats/dose group, so that the morphometric data from those rats not having homologous sections can be discarded”. In other words, exclusion of measurements from non-homologous brain sections is part of the DNT design to protect reliability of the group mean data.

Readers unfamiliar with DNT studies may not understand the unique quality controls that fortify the morphometry data in those studies. Morphometric data can only be obtained on homologous sections. Homologous sections are sections between animals that are taken at precisely the same three areas of the brain and at precisely the same angle. Aside from strict standards for section homology, it needs to be pointed out that the pathologist is blinded as to dosage group for all steps involved in selecting tissues and obtaining morphometric measurements. Therefore, very high standards are associated with allowing any single data point to be included into the highly selective morphometry data base, and including 40 of the 80 possible data points in our morphometry is evidence of the laboratories adherence to extremely strict standards.

As acknowledged in the DNT Study Report for chlorpyrifos-methyl, there was insufficient data (i.e., less than 6 individual data points per measurement) to evaluate the height of the cerebellum in PND 72 control females and the base of cerebellar lobule 9 in control and treated females. Even with few individual measurements for the height of the cerebellum in the control females at PND 72, we had the option to combine these data with the control males to bolster statistical power to evaluate cerebellum height. When males and females were combined for height of cerebellum, there were 10 control animals and 13 high dose animals with a highly reliable measurement for height of cerebellum from homologous sections. Combining males and female data for statistical purposes is an approach condoned by Garman [9] in these technically difficult DNT studies. Since i) we could combine male and female measurements to evaluate control vs treated animals; and ii) one measurement of cerebellum height is sufficient to analyze this part of the brain [2, 9, 21], the author of the study has sound basis to state that “the absence of these few data points affecting cerebellum of females at PND 72 does not affect the interpretation of the study.”

We conclude that while Mie et al. [15] use the chlorpyrifos-methyl DNT study to point out the shortcomings of a DNT study, Mie et al. [15] actually highlight the strengths of the DNT study. When a pathologist is in control of selecting the highly homologous sections in a blinded fashion, assuring that microscopic measurements only be taken on ideal sections, and has the ability to discard unreliable measurements that could otherwise obscure a test article-related effect, the study results and conclusions become most dependable.

### Proof of exposure to pups

For chlorpyrifos-methyl, Mie et al. [15] noted, *“Specifically, the study report indicates that pups were exposed via the milk, while nursing [8]. However, no data on the actual exposure of nursing pups are provided.”*

The exposure of pups to the test material via lactation must be established prior to the definitive DNT study. Data to confirm lactational transfer of chlorpyrifos-methyl are contained in the pilot study report. These data were generated prior to the DNT study because if the test substance is not transferred through maternal milk, direct dosing of the pups (generally starting around postnatal day 10–11) may be required. Thus, lactational transfer data impact the DNT study design and must be determined prior to initiation of the DNT study. The chlorpyrifos-methyl pilot study report containing (and confirming) lactational transfer data was submitted to the USEPA EPA.

### Positive control inclusion

Mie et al. [15] contend that “*test facilities are required to include positive control studies in DNT study reports, demonstrating their proficiency to correctly identify effects of known developmental toxicants.”*

Over the past 25 years, the laboratory that conducted the chlorpyrifos-methyl DNT study (WIL Research) has conducted 30 DNT studies. The laboratory has conducted proficiency testing for all behavior testing (i.e., FOB, motor activity, auditory startle, learning and memory, and neuropathology). These data have been submitted and accepted by the USEPA.

### IQ loss from exposure

Mie et al. [15] state, “*Thus, a recent study calculated the annual costs to EU populations at €146 ($171) billion from IQ losses due to chlorpyrifos and other organophosphate exposures during pregnancy [12].”*

Many experts do not agree with these health costs assessments (e.g., Trasande et al., [19]) for purported endocrine disrupting chemicals. One major factor contributing to the “highly presumptive” nature of these estimates is that the authors did not apply a weight-of-evidence approach to the epidemiological evidence and did not include studies which found no link between pesticides and neurobehavioral effects.

An independent review of the methodology and assumptions used for these cost estimates was conducted by Bond and Dietrich [3], who determined that there were “substantial flaws in the approach taken and the conclusions that were drawn”. As pointed out by Bond and Dietrich, the authors assumed a causal relationship between putative exposures and selected diseases, such as “loss of IQ” or “intellectual disability”, despite not having established causation through a weight of evidence assessment of the underlying animal toxicology and human epidemiology evidence. “Consequently, the assigned disease burden costs are highly speculative and should not be considered in the weight of evidence approach underlying any serious policy discussions serving to protect the public…”.

Bond and Dietrich also point out that numerous other, more comprehensive reviews have concluded that it is highly unlikely that the current level of chlorpyrifos exposure would have any adverse neurodevelopmental effects [4, 8, 11, 16, 18]. These studies appear to have been omitted from the Bellanger et al. [1] assessment cited by Mie et al. [15].

Mie et al. [15] note that “Independent science and regulatory safety evaluations have different purposes and speak, to some extent, different languages.” We would agree with this statement and specifically note that academic studies are often conducted under very different conditions than regulatory-required studies conducted under good laboratory practice (GLP). All studies used for registration of a pesticide must be conducted according to strict study designs and guidelines and all data are submitted to regulatory authorities who have the ultimate decision-making power as to their acceptability and scientific rigor. In the case of the chlorpyrifos DNT study, this study was conducted according to USEPA guidelines in a laboratory qualified to conduct GLP studies and the study has been reviewed and accepted by global regulatory authorities including the USEPA, Australia APVMA, Canada PMRA and the EU. The study was published in the peer-reviewed scientific literature in 2000 [14]. Labeled uses of chlorpyrifos rest on five decades of experience in use, health surveillance of manufacturing workers and applicators, and more than 4000 studies and reports examining the product in terms of health, safety and the environment. No pest control product has been more thoroughly evaluated.

## References

[1] Bellanger M, Demeneix B, Granjean P, Zoeller RT, Trasande L (2015). Neurobehavioral deficits, diseases, and associated costs of exposure-disrupting chemicals in the European Union. J Clin Endocrinol Metab.

[2] Bolon B, Garman R, Jensen K (2006). A ‘best practices’ approach to Neuropathologic assessment in developmental neurotoxicity testing – for today. Tox Pathol.

[3] Bond GG, Dietrich DR (2017). Human cost burden of exposure to endocrine disrupting chemicals. A critical review. Arch Toxicol.

[4] Burns CJ, McIntosh LJ, Mink PJ, Jurek AM, Li AA (2013). Pesticide exposure and neurodevelopmental outcomes: review of the epidemiologic and animal studies. J Toxicol Environ Health.

[5] Dobbing J, Paoletti R, Davison A (1971). Undernutrition and the developing brain: the use of animal models to elucidate the human problem. Advances in Experimental Medicine and Biology, Chemistry and Brain Development.

[6] Dobbing J, Sands J (1971). Vulnerability of the developing brain. IX. The effect of nutritional growth retardation on the timing of the brain growth-spurt. Biol Neonate.

[7] Dobbing J, Sands J (1979). Comparative aspects of the brain growth spurt. Early Hum Dev.

[8] Eaton DL, Daroff RB, Autrup H, Bridges J, Buffler P, Costa LG, Coyle J, McKhann G, Mobley WC, Nadel L, Neubert D, Schulte-Hermann R, Spencer PS (2008). Review of the toxicology of chlorpyrifos with an emphasis on human exposure and neurodevelopment. Crit Rev Toxicol.

[9] Garman RH, Li AA, Kaufmann W, Auer RN, Bolon B (2016). Recommended methods for brain processing and quantitative analysis in rodent developmental neurotoxicity studies. Toxicol Pathol.

[10] Gopinath G, Bijani V, Deo MG (1976). Undernutrition and the developing cerebellar cortex in the rat. J Neuropath and Exptl Neurology.

[11] Li AA, Lowe KA, McIntosh LJ, Mink PJ (2012). Evaluation of epidemiology and animal data for risk assessment: chlorpyrifos developmental neurobehavioral outcomes. J Toxicol Environ Health Part B.

[12] Lowe ER, Poet TS, Rick DL, Marty MS, Mattsson JL, Timchalk CA, Bartels MJ (2009). The effect of plasma lipids on the pharmacokinetics of chlorpyrifos and the impact on the interpretation of blood biomonitoring data. Toxicol Sci.

[13] Mattsson JL, Maurissen JPJ, Nolan RJ, Brzak KA (2000). Lack of differential sensitivity to cholinesterase inhibition in fetuses and neonates compared to dams treated perinatally with chlorpyrifos. Toxicol Sci.

[14] Maurissen PJ, Hoberman AM, Garman RH, Hanley TR (2000). Lack of selective developmental neurotoxicity in rat pups from dams treated by gavage with chlorpyrifos. Toxicol Sci.

[15] Mie A, Ruden C, Grandjean P. Safety of safety evaluation of pesticides: developmental neurotoxicity of chlorpyrifos and chlorpyrifos-methy. Environ Health. 2018;17(77). 10.1186/s12940-018-0421-y PMC6238321 30442131

[16] Ntzani, E.E., Chondrogiorgi, M., Ntritsos, G., Evangelou, E., and Tzoulaki, I. (2013). Literature review of epidemiology studies linking exposure to pesticides and health effects. EFSA Supporting Publication EN:497, pages 159. 10.2903/sp.efsa.2013.EN-497

[17] United States Environmental Protection Agency. Health Effects Test Guidelines OPPTS 870.6300. Developmental Neurotoxicity Study. EPA712-C-96-239. August, 1998.

[18] Prueitt RL, Goodman JE, Bailey LA, Rhomberg LR (2011). Hypothesis-based weight of evidence evaluation of the neurodevelopmental effects of chlorpyrifos. Crit Rev Toxicol.

[19] Trasande L, Zoeller RT, Hass U, Kortenkamp A, Grandjean P, Myers JP, DiGangi J, Bellanger M, Hauser R, Legler J, Skakkebaek NE, Heindel JJ (2015). Estimating burden and disease costs of exposure to endocrine-disrupting chemicals in the European Union. J Clin Endocrinol Metab.

[20] Tukey JW, Ciminera JL, Heyse JF (1985). Testing the statistical certainty of a response to increasing doses of a drug. Biometrics..

[21] U.S. Environmental Protection Agency. 1998. Health Effects Test Guidelines OPPTS 870.6300, Developmental Neurotoxicity Study. Accessed January 1, 2015 from Office of Chemical Safety and Pollution Prevention (OCSPP) Harmonized Test Guidelines, Group E-neurotoxicity Test Guidelines. http://www.epa.gov/ocspp/pubs/frs/publications/Test_Guidelines/series870.htm.

[22] WIL-406032. A Dietary Developmental Neurotoxicity Study of Chlorpyrifos-Methyl in Rats. Dow Agrosciences. LLC. July 2015:30.

