# Correction to: Letter to the editor regarding “safety of safety evaluation of pesticides: developmental neurotoxicity of chlorpyrifos and chlorpyrifos-methyl” by Mie et al. (environmental health. 2018. 17:77)

**DOI:** 10.1186/s12940-019-0489-z

**Published:** 2019-05-14

**Authors:** Daland R. Juberg, Alan M. Hoberman, Sue Marty, Catherine A. Picut, Donald G. Stump

**Affiliations:** 1 Human Health Science Policy, Corteva Agrisciences, Indianapolis, IN USA; 2 Global Developmental, Reproductive and Juvenile Toxicology, Charles River Laboratories, Horsham, PA USA; 3 Toxicology & Environmental Research and Consulting, The Dow Chemical Company, Midland, MI USA; 4 Charles River Laboratories, Durham, NC USA; 5 Charles River Laboratories, Ashland, OH USA

**Correction to: Environmental Health**

**https://doi.org/10.1186/s12940-019-0454-x**

Following publication of the original article [1], the authors reported a change in the ‘Competing interests’ section as described below:

‘Competing interests’ in the original article.

The authors declare that they have no competing interests. All willingly contributed their knowledge and perspective on the conduct and interpretation of two toxicology studies for chlorpyrifos and chlorpyrifosmethyl, respectively.

Revised ‘Competing interests’.

At the time of submission, Drs. Juberg and Marty were employed by Dow AgroSciences LLC and The Dow Chemical Company, respectively. Dow AgroSciences is the primary registrant of chlorpyrifos and chlorpyrifos-methyl in the U.S., and The Dow Chemical Company is a manufacturer of both chemicals. All other co-authors are not affiliated with either company. All co-authors willingly contributed their knowledge and perspective on the conduct and interpretation of two toxicology studies for chlorpyrifos and chlorpyrifos-methyl.
